# Poverty of Medical Men: How to Eke out a Livelihood

**Published:** 1844-02

**Authors:** 


*Poverty of Medical Men: how to eke out a livelihood.*—"One of the most certain signs (says a writer in the French Medical Gazette) of the 'decadence' of a profession, is the necessity which many of its members experience to seek for supplementary resources. Medicine is a most noble profession, but in the present day it is certainly one of the worst trades (industries) going. It is all very well to talk of dignity and high feelings; let it be remembered that a doctor has a stomach as well as a heart to attend to. In Paris alone, there are not fewer than 2,000 medical men, and it may be readily supposed that a great number of them scarcely ever get a fee. How, then, are they to live until practice comes?—by turning their talents, whatever these may be, to the best account. The other day I called on a confrère, whose circumstances I knew to be in not a very flourishing condition; I found him busily engaged in painting a portrait; he said that he had sold several of late. But what is your object? said I—'renforcer le metier,' was his answer. If a patient called, he put down his palette, doffed his blouse, slipped on his black coat, and with a grave and becomingly professional countenance, went to his consultation-room; no sooner was the interview over, than he returned to his studio, resumed his working dress, and set to his painting again with all the cheerfulness of a brave heart. 'I could not help saying, Apollo is god of the arts as well as of medicine.'

"Several young medical men have exerted themselves with success in the way of 'inventions industrielles;' for example, in the manufacture of portable liquid gas, in the construction of a night-telegraph, in preserving timber for ship-building, &c.

"The lamp C———, we owe to the ingenuity of an intelligent physician, who is practising in the suburbs, and who thus diffuses both light and health around him.

"Not a few imitate the example of *Boerhaave*, *Pinel*, and others, and give instructions in languages, mathematics, &c., translating, perhaps, Virgil and Horace in the morning to one set of pupils, and Hippocrates and Celsus in the evening to another. Often the wife, too, lends a helping hand 'à renforcer le metier,' either in the way of teaching, letting lodgings, or perhaps keeping a little magazine of linen, or silk goods, &c.—whatever, in short, that brings grist to the mill!"—*Medico-Chirurgical Review*, for July, 1843.

